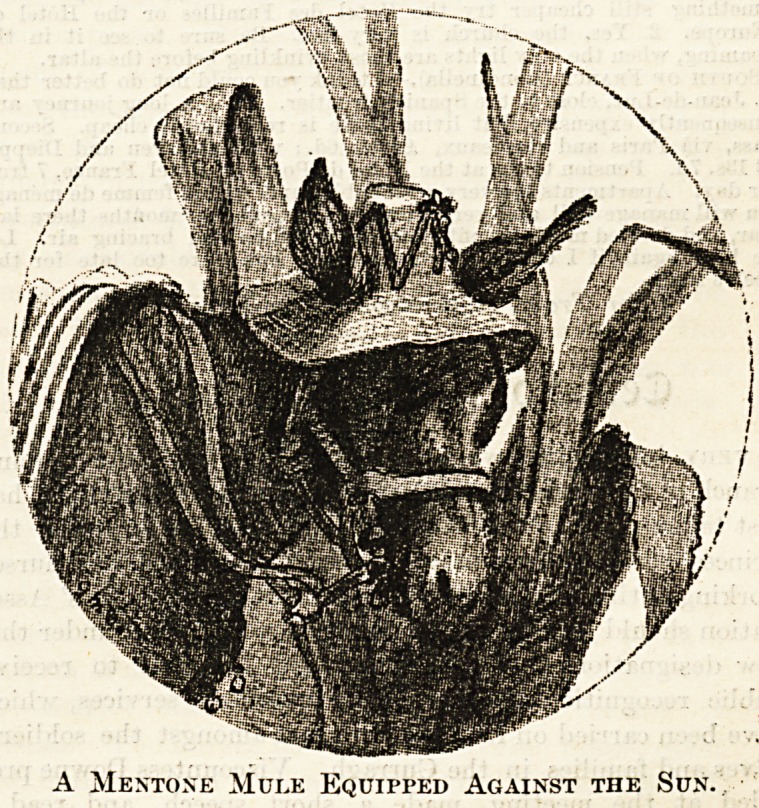# "The Hospital" Nursing Mirror

**Published:** 1899-04-22

**Authors:** 


### "The Hospital" Nursing Mirror.

Being the Nursing Section of "The Hospital."

[Contributions for this Section of "The Hospital" should be addressed to the Editor, The Hospital, 28 & 29, Southampton Street, Strand, London, W.C., and should have the word "Nursing" plainly written in left-hand top corner of the envelope.]

### Notes on News from the Nursing World.

### THE ROYAL MILITARY HOSPITAL AT ATHENS.

On Thursday, April 7th, H.R.H. the Duchess of Sparta reopened the Royal Military Hospital, Athens. The King and Queen were present, also the Duke of Sparta, and other members of the Royal Family. They were received by the Prime Minister, the Minister of War, and a deputation from the War Office, including the principal military doctors. A short service was held in the entrance-hall, and the building was consecrated by the Archbishop, who walked through the wards chanting, and sprinkling the floors with holy water shaken from a bunch of white flowers. The hospital is a fine building, entirely renovated, with accommodation for 150 patients. The floors are black and white imitation marble throughout; the wards, oil-painted pale green, are light and airy, and on one side command a view of sea and mountains. The beds are from England; and the lockers, white iron frames with three opaque glass shelves, from Germany. The wards are to be worked by orderlies under the direction of English Sisters—no easy matter, as in spite of the six months' technical teaching undergone by the orderlies before entering the wards there is much to be desired; and cleanliness, as we understand the word, is unknown. The hospital is under the patronage of the Duchess of Sparta, who takes a lively interest in all nursing matters.

### NURSES FOR WORKHOUSE HOSPITALS.

It is encouraging to learn from the eighth annual report of the Northern Workhouse Nursing Association that 1898 was another year of continued activity in the training and supplying of nurses for workhouse hospitals. During the twelve months there were 54 applications from boards of guardians, and 37 nurses—of whom 32 were trained by the association—were supplied to various unions in both the northern and southern districts. The committee express their regret that they are unable to keep pace with the demands made upon them for trained nurses, though there are now 45 workhouse hospitals, chiefly north of the Trent, employing 77 nurses supplied by the association. An interesting passage in the report is to the effect that the work of the association nurses is more appreciated each year by boards of guardians. The prejudice formerly entertained by most union officials against a "trained nurse" is fast disappearing, as the advantages which a trained nurse possesses over an untrained one are proved to them by experience. It is also stated that "as a rule much kindness is shown to our nurses by the masters and matrons under whom they work." Of course, there is occasional friction between the untrained matron and the trained nurse. As to the failure of an ample supply of trained nurses, we agree with Dr. Rhodes, the chairman of the association, that "if boards of guardians would establish for nurses comfortable apartments, fair salaries, and reasonable conditions of service, there would be no lack of well-trained, intelligent, and sympathetic nurses."

### AN ANGLO-AMERICAN NURSING HOME FOR ROME.

No one who has stayed in Rome for any length of time can require to be assured of the urgent need of a nursing home for English-speaking persons in that city. In the event of visitors falling ill at their hotel apartments it is difficult to obtain, under present conditions, the attendance of a skilled nurse, and accommodation at a suitable home is out of the question. The efforts which are now being made to supply a much recognised want therefore merit the warmest support. An influential committee, with the British Ambassador as president, Sir George Bonham as vice-president, including among the members the Bishops of Gibraltar and Southwark, Dr. Wilkinson, Lady Vivian, Miss Brabazon, and Mrs. R. Crawshay, has been formed; and it is proposed to hire a detached house, which will be comfortably furnished. Patients of every denomination will be received, and attended by their own doctors and spiritual advisers; a staff of trained nurses will be maintained, and the latter will be available, if needed, to attend patients outside. We are glad to notice that there will be several free beds in the home for patients who may be unable to contribute anything to their own maintenance. The charges for attendance to others will be on a sliding scale. There is no reason why the institution should not become self-supporting in a short time; but at the outset some financial assistance is needed, and contributions for the treasurer, Mr. E. F. Ede, can be paid in London through Messrs. Coutts and Co., 59, Strand.

### THE NEW NURSING QUARTERS AT THE SUSSEX COUNTY HOSPITAL.

The formal opening of the new quarters for the nurses at the Sussex County Hospital will take place on Saturday, May 13th. Sir Henry Harben, an ex-president of the hospital, has kindly consented to preside. The building consists of a handsome structure detached from the hospital, but in the hospital grounds, and is connected with it by a covered way nearly 200 yards long. There is accommodation for about 50 nurses. The house contains besides the 50 sleeping rooms (not cubicles) two good sitting-rooms, a sick room, a kitchen, four bath rooms, box and linen room, and an office for the use of the acting home sister. It is lighted throughout by electricity and heated by hot-water. Standing on a height far above the hospital, it not only has the advantage of fresh air, but the views of the Channel and surrounding country are most beautiful. The quarters for the night nurses are at the top of the building and are entirely shut off from the other part to prevent unnecessary noise. Care has been taken to provide ample escape from all parts of it in case of fire by means of two iron staircases outside the building and opening on to either corridor. It is a pity that there were no iron staircases outside the Hyde Park Court flats, which were partly destroyed by fire this week. The cost of the quarters, which will amount to nearly £5,000, not including the furnishing, has been largely provided for by the kindness of three most generous friends who are much interested in the hospital, and a portion of the money raised in Brighton in commemoration of the Queen's Jubilee has also been assigned towards the payment of the expenses.

### ESSEX COUNTY COTTAGE NURSING ASSOCIATION.

The annual meeting of this association was held at the Three Cups, Colchester, on Tuesday afternoon, the Mayor presiding. A large number of persons accepted the invitation of Lady Rayleigh, the president, and the members of the committee. The association was founded five years ago, to train and supply cottage nurses for the country, and to assist in the organization of local associations for supervising the work of the nurses, and collecting and guaranteeing their salaries. The training home is at Leytonstone, and has lately been enlarged in order that a greater number of nurses may be trained to meet the increasing demand.

### THE INTERNATIONAL CONGRESS OF WOMEN.

There is some danger that this gathering, which will be held in the Queen's Hall on June 27th and two following days, will attempt to do too much. The programme appears to embrace every possible subject, and there are no less than five sections. Special prominence is given to the Political Section, in which it is stated that the strength of the United States will be felt. In our judgment the Political Section is the least important. Much more interest attaches, for example, to the Professional Section. In this department Mme. Marie Jopelin, who is a member of the Bar in Belgium, will read a paper on the professions open to women in that country; and Mrs. Hampton Robb, president of an American Nursing Association, will read an important paper.

### "THAT DREADFUL CHURCH BELL."

It is, no doubt, necessary that the church bell should proclaim the hour for Divine service. But it need not be cracked, and it need not go on with the proclamation for a quarter of an hour. Very reasonable complaints are made by the authorities and inmates of the nursing homes in the vicinity of Marylebone Road concerning the extremely unmusical bells of a neighbouring church, which are set in motion every Sunday four times a day, and toll continuously for 15 minutes. It is possible that the funds at the disposal of the vicar and churchwardens will not permit of the purchase of new bells; but they would earn the gratitude of many poor souls suffering pain and urgently requiring all the rest they can obtain, if they would reduce the period of tolling or ringing. Five minutes of the "music" would, surely, suffice to remind the parishioners that the hour of worship is at hand.

### ST. GEORGE'S BOMBAY.

Much credit is due to the hon. secretary of St. George's Hospital, Bombay, for his energetic endeavour to place the nursing institution in connection with it on a firm financial basis. This hospital, which is European, contains 172 beds, and, apart from the nursing, is maintained by Government. Moreover, the Government contributes Rs. 10,000, or about one-third of the cost of the nurses. The remainder of the income is derived from the profits of the private staff; interest on an endowment fund; a valuable contribution from the Poor Trust, amounting to a total of about Rs. 15,000; and public subscriptions of about Rs. 5,000. The nursing is under the superintendence of the Sisters of All Saints, who number 27, but the actual work is performed by 36 nurses and probationers. The Sisters, however, supply the places of the lay nurses when any are absent. The extra nurses are accommodated in cubicles in the free wards of the hospital, and it is desirable that the new quarters, which are now the subject of negotiation with the Government, should be built as soon as possible. It is hoped that the work will be begun before the monsoon.

### NURSES IN THE CITY OF LONDON.

Some little stir has been caused in the City by the scheme of Dr. Sedgwick Saunders for an isolation hospital. At present there is only a shelter in Cotton Street for poor patients suffering from infectious diseases, and this is notoriously inadequate. Besides, the premises are no longer available, and the time has arrived for deciding whether new premises should be erected in Golden Lane. The plan includes rooms on the first floor for patients suffering from disease, and sleeping accommodation or shelter for families displaced by the outbreak of disease under circumstances which render the removal of the patient impossible. On the second floor, ten beds will be provided for each sex, and the third floor is to be held in reserve for further hospital accommodation. No nurse who is familiar with the existing shelter will fail to recognise the importance of improved accommodation for City patients.

### SCARLET FEVER CONVALESCENTS.

An effort is being made to raise £2,000 in order to make good the loss occasioned by the fire last Whitsuntide at the Mary Wardell Convalescent Home, Stanmore, the insurance being insufficient. This is the single institution that receives patients recovering from scarlet fever, except those under the Metropolitan Asylums Board. Poor persons only pay from 10s. a week, but ample provision is made for the more well-to-do, from three to five guineas a week. Funds are also needed to defray the initial cost of connecting the drainage of the establishment with the public sewer, recently made, as well as to put everything in a state of thorough repair and cleanliness. The number of inmates necessarily varies in proportion to the prevalence of the disease, but the fixed expenses remain stationary. The fact that the income shows a steady, if slight, increase during the past five years is satisfactory, but there is room for further improvement in this respect.

### THE MIDWIVES BILL.

This measure was the third order of the day in the House of Commons last week, and the failure to secure the second reading stage then is fatal to its chance of becoming law this session. The Manchester Midwives' Society lately passed a resolution in opposition to the Bill, but a much more important organisation, the Midwives' Institute, takes a different view of the matter, and would be glad of legislation which would improve the present highly unsatisfactory state of afairs. It is, however, these differences of opinion among people directly interested that provide

Members of Parliament with reasons for slackness in supporting the movement for the amendment of the law. For years the plumbers have been vainly trying to get a Registration Bill through the House of Commons, because of the want of unity among themselves; and their experience should not be lost on midwives.

### HONOURS FOR NURSES.

The Queen of the Hellenes has graciously bestowed a medal and diploma upon the English nurses who took part in nursing the wounded Greeks in the late Greco-Turkish war. The following ladies have received this honour in recognition of their devotion to duty: Mrs. Bedford Fenwick (superintendent of nursing, National Fund for the Greek Wounded), Miss Isabel Carter, Miss Jane Child, Miss Sarah Collins, Miss Isabella Coombs, Miss Emma Curtis, Miss Amy Davidson, Miss Alice Davies, Miss Emma Dobson, Miss Beatrix Farnsworth, Miss Lavinia Fawkes, Miss Charlotte Flanagan, Miss Emily Fox, Miss Clara Hill, Miss Gertrude Johnstone, Miss Annie Latham, Miss Lilian Lees, Miss Margaret Moody, Miss H. C. Nisbet, Miss Jessie S. Parson, Miss Katherine Stollard, Miss Florence Skerman, Miss Ellen Tillott, Miss Kathleen Waller, Miss Kate Walker, Miss Lillie Warriner, Miss Henrietta Whitegord, and Miss Alice Winder, all nurses of the National Fund; Mrs. Ormiston Chant (Cretan Nursing Fund); and the following volunteers: Miss Palmer, Miss Dunbar, Miss Wallace, Miss Head, Miss Lavinia.

### THE PRINCESS FREDERICA'S HOMES.

Nurses who are no longer as young as they used to be, and whose private income is of too small a character to feel that the comfort of their declining years is assured, will be glad to be reminded of The Homes for Gentlewomen, Warwick House, Tulse Hill, which provides an asylum for a limited number of ladies over 60 years of age. Each inmate must be possessed of some means; and then she is provided with the use of an unfurnished room and a common sitting room. She may also make arrangements, if she wishes, to have her dinner sent to her daily from the superintendent's table. The Committee of Management only pledge themselves to supply house rent, gas and coals; but medical advice is likewise given free by a firm of local doctors. The cost last year was £599, whilst the subscriptions amounted to only £315. This state of affairs compelled the committee to use money for the purpose of maintenance which they would have been glad to invest. The floral fête last July was most successful in spite of the regretted absence of the royal patroness, Princess Frederica. It resulted in the welcome addition of £192 to the funds of the institution. This entertainment will be combined, for the future, with the sale of work, and will take place annually.

### THE ROYAL ROAD TO NURSING.

Nurses who have experienced the arduous initiation into the art of nursing considered necessary in this country will be of opinion that the high-born damsels of Germany have discovered for themselves a Royal road to nursing. The Princess Elizabeth von Hohenlohe is the originator of a new society for nursing the wounded in war. She is the Chancellor's daughter, and has gathered round her about twenty ladies of rank, who voluntarily attend, and undergo, courses of theoretical instruction at the Wilhelm's Akademie, the military medical school at Berlin. Practical demonstrations are given in the accident wards, and the student has a four weeks' spell in a garrison hospital. The Empress is keenly interested in the movement, and paid a surprise visit to the Akademie the other day.

### THE CEYLON NURSES' ASSOCIATION.

The annual meeting of the Ceylon Nurses' Association was held last month at the Adam's Peak Hotel, Hatton, Mr. F. G. A. Lane presiding. The report showed a not unfavourable advance as compared with that of previous years, but the reorganisation of the system of account-keeping rendered some explanation from the treasurer necessary before the fact was apparent. The record of the work indicates that the services of the matron (Miss Shankland) and her staff of five nurses are much appreciated, and that more nurses are wanted. The number of cases attended during the year was 45, and four in the wards adjoining the home. Quite a formidable code of new rules was submitted by the Executive Committee and accepted. The rules are in two portions; the first relates to the payment and regulations relating to the employment of the nurse; and the second to her duties, dress, rest, &c. They are very definite, afford little scope for misunderstanding, and seem reasonable, excepting that which gives a nurse only eight hours' rest and recreation in the twenty-four. To make the matter worse, four only of these are of necessity consecutive. How can any nurses possibly enjoy tolerable health or do their work efficiently with but four hours' unbroken sleep?

### SHORT ITEMS.

The Watford Guardians have supplied the children under their charge with tooth brushes, and care is to be taken to number each brush and place it in a rack. A lady guardian suggests that there should be "a tooth-brush drill" each evening, and it is obviously of importance that, having very properly been given the brushes, the children should know how to use them.—The Rev. Dacre Craven, who has just been preferred by the Duke of Buccleuch to the important post of rector of St. Andrew's, Holborn, has been hon. secretary of the Metropolitan Nursing Association for the past 18 years. It is hoped that in his new sphere of duty Mr. Craven will still be able to continue his connection with the association.—In the district of Mr. H. Jenner-Fust, which includes Lancaster, part of Cumberland, Westmoreland, and part of Yorkshire, the average number of patients to each nurse is 13. The actual number of nurses on day duty is 510; on night duty, 154; whilst that of the sick is 9,084.—The crowded-out nurses of the Parish Street Workhouse, St. Olave's, occupy certain cottages in Parish Street, and are without supervision. The Local Government Board inspector suggested a bridge to connect the workhouse and the cottages, in order that the matron might be enabled to exercise greater control; but the Guardians, we regret to observe, do not see their way to constructing it.—In aid of the Children's Ward of the Passmore Edwards Hospital for Willesden, a very interesting and successful café chantant was given under the direction of Mrs. St. Hill, on Saturday afternoon, in the Portman Rooms, Baker Street.

### Antiseptics and Operations.

A Lecture delivered to the Nurses of the Victoria Hospital, Hull, by Alfred Parkin, M.S., M.D. (Lond.), Senior Surgeon to the Hospital.

Since operations are things that occur daily in every hospital it is most essential that all nurses should have some idea of the way in which they are carried on, and why they are carried on in particular ways. At one time in connection with operations dressings were considered of minor consideration. Forty years ago, after removal of a leg, it was simply the practice to put on a poultice and to change this poultice as often as was necessary, perhaps every four hours. The result was that the stump after a time began to suppurate and form pus, and it was quite impossible for a wound of that description ever to heal up as wounds do at the present day. Probably you know that after an amputation nowadays everything is sewn up, and after a week the stump is practically healed. That is, of course, what one calls a good result, and which one ought to obtain every time such an operation is performed.

But formerly such results were never attained. At a time more remote than that I have spoken of the stump would be dipped into a bucket of boiling tar, and I am not sure that the practice was not rather better than the poultices.

But we have to consider to-day a much more scientific method, and I want to give you the reasons why such methods are adopted. You have all, no doubt, been at the hospital some time, and have been told what to do in the operating theatre and in the wards, in the way of getting dressings ready, and have also seen dressings applied to patients. But you have possibly no idea why all this is done, and so I am going into the subject with as little scientific detail as possible, because I am sure if one gave you too much theory you would get very little that was practical.

Now you know there are such things as germs, and it is these germs which enter into most of your work on the surgical side of the hospital. Germs are of three kinds—micrococci, bacilli, and bacteria. I will write the names of them on the board, as it would be well for you to see how these words are spelt, for if you ever have to write them down, do try and write them down properly.

Micrococci are extremely small; say if I made 20 or 30 dots on the board, they would represent 20 or 30 micrococci, because they are round and small; they are, in fact, minute little spheres. To see them you have to have a microscope of high magnifying power; you have to magnify them a thousand times before you can see them at all, so you can understand it is utterly impossible ever to see them with the naked eye, and it is not always very easy to see them with the microscope. Now, these micrococci are the active agents in the formation of pus, so they are most important, for if anything does go wrong with a wound the first thing it does is to form pus. There are numerous forms of micrococci, one of which produces pneumonia and another diphtheria. These few details are enough for you to remember at present, because later you will have given you names of different diseases which depend on these micro-organisms.

And now we come to bacilli, which are of rather a different form. If I draw a few short lines on the board they will resemble the appearance of bacilli. But these marks are about a million times bigger than bacilli really are. You require a very powerful microscope indeed to see them and recognise them. One of the diseases they are concerned in is phthisis or consumption, and you will find them in millions of millions in the lungs o patients who have consumption.

Then finally we come to bacteria, and these are much the same as bacilli, only they have thick bodies and their length is not quite double their breadth; simply remember they are stouter and thicker than bacilli, and that they enter into all forms of decomposition. When you get nasty smells, or decomposition of flesh, or decomposition of dressings, there you find heaps of bacteria and other micro-organisms, if you only examine with the microscope. Of course there are other ways of finding these things, but I think what I have told you is sufficient for our purpose to-night.

And now where are these organisms found besides? I have told you the diseases in which they are found, but then that is not everything. You might say, for instance, that this table before us is beautifully clean. It looks so, and I have not the least doubt it is. But if I were to rub a bit of cotton-wool over the table and then examine it under the microscope, I have no doubt we should be able to discover hundreds of germs which came from a table that looked perfectly clean. In fact, these little bodies are all over you. You have thousands upon thousands in your mouths, and millions beneath your finger-nails. So you see they are practically everywhere, and you will understand by this how these germs come into the question of operations. For instance, you make a cut in a child's body—perhaps you open up the body. There are germs in the air, possibly in the hands of the nurse who is helping you, possibly in your own hands. And if any of these germs get into the child's body there is a risk of dreadful things happening—for one thing, they can cause peritonitis, a disease frequently leading to death in a couple of days.

If you have a patient on your hands with consumption, and you are not careful in the disposal of the stuff he coughs up, that stuff, or sputum, dries up as it is exposed on handkerchiefs, and passes into the air; perhaps you inhale it yourself, or other people in the house do, and so consumption passes on from one to another. Similarly if you allow cast-off dressings or soiled diapers after a confinement to lie about, you get a smell indicative of a tremendous number of micro-organisms existing on those dressings, and these germs can be blown on to other patients with very serious results.

"Well," you will say, "how is it possible to do any operation successfully if these germs are about and trying to get a foot-hold, and so prevent your wound healing up satisfactorily?" You may take it for granted that any wound made in a person who is not a subject of an abscess or of septic disease should heal up in the course of a very few days without the formation of any matter. If you get a cut finger and it does not heal up without festering you may be sure you have got that finger poisoned; it may be poisoned in a very mild way, or to such a dreadful extent as to give you blood-poisoning of the arm. I knew a nurse very well who laid out the body of a patient who had died from a septic disease, and, unfortunately, she pricked her finger with a pin, and died at the end of a week from very acute blood-poisoning. I do not say she was a very careful nurse—she was not. And this shows you it is just worth while considering whether the taking of a few precautions will not save you a great deal of trouble and other people quite as much.

And now how to get over this germ difficulty. I can quite understand that you will argue straight away that we are all alive, and that germs do not seem to affect us very much. This is not quite true, because many die every day from germ diseases, but there are a great number of people living, and there must be some way of rendering these germs harmless.

*(To be continued.)*

### Wants and Workers.

Could anybody, writes the District Nurse, The Home, Long Ashton, Bristol, kindly let me have a second-hand perambulator for a poor woman who has lately had twins? She cannot afford to buy one and cannot get out at all with the two babies till she has got a perambulator for them. If anybody would kindly help in the matter I shall be glad to know.

### Post Graduate Clinics for Nurses.

### THE USES OF WATER IN THE SICK ROOM.

### III.—Packs and Vapour Baths.

The wet sheet pack is of time-honoured observance in the treatment of acute febrile disease and in some forms of chronic disease, and since its application is so general I will content myself with a few practical hints rather than a detailed description of the methods whereby it is applied.

A very large sheet is necessary, since this ought to be sufficiently capacious to wrap twice round the patient's body. Three blankets should be placed on the bed, and the end of the sheet which the nurse holds while wetting it should be gathered into accordion pleats, so that it will be very easy to spread it out when thoroughly saturated. Cold water is rarely ordered. 100 deg. F. is a good temperature, since some eight to ten degrees will be lost in the wringing. It is important to wrap the sheet and blankets round the patient in such a manner as to keep out the air. The blankets at the feet should be long enough to cover them and tuck well underneath. The nurse must, however, carefully avoid swathing the pack too tightly, since nothing can be more uncomfortable to a patient than to be bound down by such restriction, and she must watch for any coldness of the feet, in which case a hot bottle should be applied to them. If the patient does not become warm in ten to fifteen minutes, fresh blankets should be added and artificial heat applied to the sides. In those cases where chilliness persists, the patient, removed from the wet pack, must be rubbed vigorously and placed in a dry pack. It is well to apply a cold compress to the head during the wet pack and to re-wet this compress occasionally. The duration of the wet pack depends on medical instructions. Sometimes the patient falls into a refreshing sleep, but while in such a pack he should be kept under strict observation, since faintness and exhaustion and other unfavourable symptoms sometimes supervene, which call for immediate removal from the pack. The nurse should in all cases obtain explicit instructions from the doctor as to the exact duration of the pack and the temperature of the water to be used for it. After removal from the pack a warm quickly-given sponge bath is necessary, and the patient must be carefully guarded from draught or chill.

If a hot pack is ordered, an old blanket is preferable to a linen sheet. The shower pack is used in some cases of extremely high fever accompanied by delirium. The patient is placed on a bed which is protected by a rubber sheet; he is wrapped in a wet pack but not covered with blankets or any covering but the wet sheet itself. Enveloped in such a pack, rapid evaporation takes place, which speedily brings down the fever. As the water evaporates the dampness and coldness of the sheet is kept up by means of cold water sprinkled from a watering-pot over the patient. Such a pack as this must never be used unless under direct medical orders, since it is somewhat heroic treatment, resorted to only when fever and delirium run dangerously high. When a patient is immersed in a cold bath for reduction of temperature he should be entirely wrapped in a sheet. In some cases it is found advisable to anoint the entire body with vaseline before putting the patient into the full cold bath, this simple precaution much diminishing the shock caused by the fevered body coming suddenly in contact with water so much cooler in temperature.

No nurse can give such a bath unassisted. In cases where the patient is not helpless, a cooling bath can be given while he sits in tepid water, the nurse throwing water gradually colder and colder over him. Such a bath may last from five to twenty minutes, or even longer, according to the effect it has on his temperature. This is what is termed affusion, and it proves most valuable in the case of delirious, hysterical, and maniacal patients. If the patient be very excitable it is best not to dash the water over him; it may be applied in more gentle and less terrifying fashion by means of a watering-pot, or better still, by means of water thrown with some degree of force into a common kitchen colander. Here we have a very fair imitation of the shower bath without the sudden shock, which makes it inadvisable for the nervous, the very excitable, or for child cases. Few children ordered this treatment object to the watering-pot or the colander. Indeed, they regard it more or less as an amusing game. This kind of affusion is ordered in some forms of convulsions and in tetanus, and it seems hardly necessary to remind the nurse that it should never be applied without medical orders.

It is a useful reminder that in all cases of prolonged baths—hot or cold—a wet compress should be applied to the head, which must be periodically cooled and wetted. Thus patients troubled with "rushes of blood" to the head are relieved, and the uncomfortable "flushings" caused to some persons by bath treatment are diminished and the chances of faintness lessened. The half-pack is sometimes used for feeble patients who are not able to bear the depressing effect of the entire wet pack. For this treatment the wet sheet extends only from the armpits to the thighs, but the blankets should still cover the entire body. This form of pack is used in lung diseases, such as pneumonia, croup, and bronchitis, and in some abdominal and pelvic inflammations. Sometimes it is applied only to the chest, and is thus used to bring down fever, or for its effect on the lungs. If either the half or the chest-pack is employed with hot water, a length of flannel or a portion of blanketing should be used. If tepid or cold water be employed Turkish towelling serves admirably.

The warm or hot vapour bath ranks among the various baths used in the sick room, and I will describe the most simple method of giving it. This bath is employed in rheumatism and as a diaphoretic in Bright's disease and other kidney trouble, in some liver cases, and to relieve or correct inactivity of the skin. There is no object in an elaborate apparatus, since a cane or wicker-seated armchair (the seat covered with a Turkish towel) serves admirably. The patient is seated on the chair, and he and the chair are entirely surrounded by a blanket reaching well down and trailing on the ground.

Over this blanket two or three more should be secured with safety pins close round the patient's throat and well over the chair. A tub underneath the chair should now have about a gallon of absolutely boiling water poured into it, the blankets being drawn close and kept in place on the ground by two or three weights, such as kitchen irons, &c. Very shortly a corner of the blanket should be raised and an extremely hot brick, held on a shovel, should be lowered into the tub. Another brick may be added in three to four minutes, and so on until the patient is steaming with perspiration. He will readily distinguish whether the moisture on his skin is perspiration or merely the result of condensed vapour. If the patient complains that the vapour is too hot, a little air may be judiciously admitted by raising the blankets an inch or two at one point.

A cold compress should invariably be applied to the head of a patient undergoing vapour bath treatment. The duration of such a bath will depend upon medical instructions. A warm sponge bath—the patient being carefully dried and kept from all possible chill—completes the giving of a vapour bath.

### To Nurses.

In order to increase and vary the interest in the *Mirror*, we invite contributions from any of our readers in the form of either a paragraph, a letter, or information, and will pay a minimum of 5s. for each contribution.

### The Ethics of Nursing.

### THE DANGER OF GROWING SLIPSHOD.

THIS danger is in the nature of things more applicable to the private than to the hospital nurse, and it is a great and growing danger. During our training, and during any after-time we may spend in hospital, our chances of growing slipshod are remote and few. In the wards of a hospital we are always being kept—willy nilly—up to the mark. Each day is mapped out for us; the work goes on with machine-like regularity; each hour has its appointed task; and we can no more drift into irregular times or habits than if we were wound up, added to which there is always someone in authority to check tendencies towards unpunctuality or inaccuracy, or any other slipshod ways.

But the case is very different when having shaken the dust of her hospital off her feet, and the yoke of hospital discipline from her shoulders, a nurse begins to do private nursing. Now comes the time when the real good of her training tells. It is the crucial test of her capabilities as a nurse when she is left to stand alone, when there is nobody in authority to pull her up for derelictions from duty, or to share responsibility. And I have noticed with what ease, as the months and years slip by, a nurse, who in hospital was up to time, accurate, smart in her work, glides into slipshod ways and unmethodical habits. This does not happen at once. Hospital training is grounded deeply enough to last for all the first months, and then the drifting process begins. There is only one patient now to be tended instead of several—very well, then, what does it matter how much that one patient is pottered over? How can it signify whether he is washed at nine, or ten, or even eleven o'clock? There are no dressers coming in as the clock strikes ten—there is no necessity for having the room and the patient smartened up by a given hour. This seems to be the line of argument of the nurse who is gradually growing slipshod. In the first place, it is always in itself a mistake to be slipshod. In the second place, if you let yourself grow careless about a non-essential, how long will it be, do you think, before you grow equally careless about essentials as well? And, thirdly, be your patient rich or poor, in a ward or in a private house, he will gain by being nursed as regularly and methodically as possible. You cannot, of course, introduce too much hard and fast discipline into private nursing, but the more you can judiciously manage, the better for the patient. Nothing is more conducive to a patient's moral and physical well-being than the sensation that clockwork regularity and punctuality are the keynotes of every day. Only a few weeks ago I was struck by the slipshod ways of a nurse I came across. She had been trained in one of the best and most important schools of the day, but the mantle of her training seemed to have dropped from her shoulders. Her nursing was of the happy-go-lucky order. She took the patient's temperature when she happened to think of it, though it is just as easy and no more annoying to the patient to have it taken at fixed hours. Medicine was given with a delicious vagueness, or was not given at all. "Oh! I say! I've forgotten your medicine all day!" was one remark I heard from the nurse's lips. There was precisely the same casual uncertainty about the feeding of the sick person. The doctor had given orders that the invalid was to be fed often—at least every two hours—in small quantities. But those little meals appeared with blissful irregularity—just when it happened to occur to the nurse to bring them. It would have been as simple, and far better for the patient, to have had the soup, or jelly, or whatever it might be, exactly every two hours, on the stroke of the clock. That nurse had a fatal habit of pottering, a habit very easily acquired in private nursing. She dawdled over the patient's washing operations, she was half the morning setting the room in order; there was an unrestful feeling that she was perpetually "on the go." It may be argued, "Ah! but private patients do not like to be hurried and to be made to do things up to time." They may not like to be hurried, and nobody wishes them to be. Still, there are very few of them who do not like work done up to time. It is most fidgeting and worrying to lie in bed and watch someone else potter round. Then it may be said, "But, after all, this is a trivial matter." Is anything really trivial? If you are slipshod in little things will you not soon be slipshod in great? Punctuality and accuracy may one day be matters of life and death. The small beginning of doing things in haphazard fashion may end presently in their not being done at all. "Oh, I never did so and so," is a frequent exclamation from a slipshod person. By and by it will be, "Oh, it doesn't matter."

But one day there will come a moment when "it will do presently, it does not matter now," cannot be said; when a serious dereliction will end in grave disaster, and a nurse may find her whole career ruined because she has allowed slipshod actions to grow into slipshod habits. Does the famous quotation seem too grand for the occasion? I think not, for when all is said, nothing in this world is too insignificant and small to have sometimes far-reaching consequences, and the great quotation is not too great even for a little fault like being slipshod. For speaking of actions and habits reminds me of these words: "Sow an act, you reap a habit; sow a habit, you reap a character; sow a character, you reap a destiny."

### Novelties for Nurses.

### PRYCE JONES (LIMITED).

INDICATIONS are not lacking that brighter days are at hand, but it may be laid down as a safe axiom that flannel and woollen garments will hold their own for some time to come. Quite the most seductive of all the patterns we have had submitted to us are those from the far-famed emporium of Pryce Jones (Limited), of Newtown, Wales. The variety and beauty of the designs this year are unequalled, and are offered at a price that brings them within reach of the least affluent of our readers. Imagine dozens of samples in striped washing flannels, so fine that they might easily pass through the proverbial ring of the fairy tale, and presenting a harmony of colour and an elegance of design that would be difficult indeed to surpass. Then there are plain molletons in every shade, suitable for dressing-gowns, and homespuns for the modest price of 6¾d. per yard that would make up into the daintiest of costumes. The serges, too, are of excellent quality, and will stand any amount of hard usage. This firm does not, however, limit its range to woollen materials only. We would specially draw the attention of our readers to the made-up goods, which are a marvel of style and cheapness. Flannel bed-jackets can be had in all sizes; men's dressing-gowns warmly lined cost only 12s. 6d. We also admired a useful wrapper or cottage gown of blue serge, made somewhat loose and confined at the waist with a band, the price complete being 6s. 9d. Nurses' aprons are another specialité, and are to be had in all shapes and sizes at prices commencing at 1s. 9d. upwards, according to quality. Servants' aprons, also, are a charming line, and one or two fancy working aprons, piped with pink cambric, are most fascinating. We must not omit to mention the more domestic articles, such as towels, which are made in the finest huckaback, and quilts of woven cotton. Nurses who are requiring cloaks would find all their wishes anticipated in the best possible style for 17s. 11d., and we strongly advise them to send for patterns without further delay. Everything sent out by this firm is of the very first quality, and it deserves the success it has so long and honourably maintained.

### Nursing on the Niger.

### A CHAT WITH ONE OF THE NURSES OF THE EXPEDITION.

SINCE the return of the nurses from the Niger last week, I have been fortunate enough to have a chat with Miss Nutt, whose pleasant, bright face shows few traces of the severe test to which her health has lately been subjected.

The question of climate was, naturally, the first topic of conversation, and Nurse Nutt admitted that "she has found it very cold here."

"That, I expect," I rejoined, "is partly by contrast. Was the heat on the Niger very difficult to stand?"

"The climate is very trying—one of the worst there is—and we suffered a good deal when we went out from fever; but, then, we were the pioneer nurses—no white women had been out there before—and nothing was in working order There was no hospital built; we occupied only a temporary building, a hut with corrugated iron roof, and had to discover for ourselves, to a certain extent, the best way to keep well and strong. Also, we landed just before the tornado season, which is about May or June, and is the most unhealthy time of the year, so that we had to work at once under the most trying conditions. After July, when the wet season began, it got cooler, and matters improved."

"Did you personally suffer much from ill-health?"

"I had repeated attacks of fever, and so did Nurse Powell; but strange to say, Nurse Clarke, who thought she would stand the climate worse than any of us, proved to be the least affected by the excessive heat."

"I suppose the fever you speak of is malarial?"

"Yes. Severe attacks sometimes prove very troublesome, but a simple attack lasts for three days, two of which at least should be spent in bed. Then you begin to pull round, though the consequent weakness is difficult to overcome. When the attack was very slight, we did not always give up work."

"You were sent out by Government?"

"Yes, by the Colonial Office. When the other nurses at Guy's heard we had volunteered and been accepted to accompany the expedition, they nearly bade us good-bye at once. They never believed that we should live to come back again."

"Were you bound by any special conditions?"

"We undertook to remain on the Niger for a year, so that with the journey to and from West Africa we were absent fourteen months from England."

"Do you think a white woman should remain longer than a year?"

"It would be most unwise to do so. Even the soldiers are granted a furlough, or a change after twelve months' service."

"I understand you were not with the moving forces, but in a permanent hospital?"

"When we went out we were not sure which way it would be, and our uncertainty as to our ultimate destination was increased owing to the possibility, then existing, of war between England and France. That fear happily passed away. We found our duty was to lie between the two hospitals, the larger one at Lakoja, the smaller at Jebba."

"Are they near each other?"

"The distance between the two is 250 miles. Lakoja is about 400 miles from the coast."

"How did you travel?"

"Entirely by water. We were fortunate enough to have a launch provided for us; otherwise we should have had to go by canoe, which is not nearly so pleasant."

"What assistance had you in the hospital work?"

"Non-commissioned officers helped us in the actual nursing, and black boys did the rough labour."

"Did you find the boys difficult to manage?"

"Oh, yes. The only way to get them to put their shoulder to the wheel was to be constantly after them. We had been told when we went that we should have to learn a little of the native language, and accordingly we took out books for the purpose. But as a matter of fact we mastered only a few words, for we found that the black boys soon picked up a sort of pigeon-English, and readily understood our orders after quite a short time."

"Of whom did the medical staff consist?"

"Of eight doctors, all English. Part of them were with us at the hospitals, others were up the river with the forces."

"What disease was most prevalent after malarial fever?"

"Black water fever, which is peculiar to the climate. It is a grave disorder. Malarial fever is largely treated with quinine. Black water fever is different, and the symptoms are much more serious."

"Are lung diseases prevalent on the Niger?"

"Not amongst the English. I do not remember having a single cold myself all the time I was away, but the natives suffer a good deal from phthisis and pneumonia. Sometimes, too, they have epidemics of small-pox."

"Did you have much night work?"

"No, not generally, unless we had an unusually severe case. The non-commissioned officers, having been so well trained in the army hospitals, are very capable, and can mostly be left in charge at night."

"Did you and your companions work together, or at different hospitals?"

"The first eight months we all spent at Lakoja; the last four at Jebba, where the hospital is built on two rocks in the middle of the river, very barren and very hot, but supposed to be healthier because of the air all round. We could not separate because one nurse could not go hundreds of miles away by herself. The expedition had applied for four nurses and hoped that they would have been sent. I believe four will go out next time, so that two can be at one hospital, and two at another. They are starting almost at once, but not nurses from Guy's."

"I should like to know what uniform you adopted?"

"White washing dresses, of the lightest material, and tan shoes. When we went out we wore no regulation bonnet, but a pale grey helmet, so as to avoid any risk of sunstroke. It is necessary always to wear flannel, however thin, as changes in the temperature are sometimes very rapid, and chills are especially to be guarded against. A warm cloak is advisable for the evenings in December. It is frequently quite chilly out of doors in that month."

"Were the insects very troublesome?"

"Mosquitoes and sand-flies abound, and nets round the beds are required. All the beds and bedding came out from England, but we used no sheets. We found very thin blankets of a special kind were far safer as a covering, reducing the risk of night chills."

"I conclude that all water and milk you used in the hospital had to be boiled?"

"We found that every drop of water had to be treated in that way, but unfortunately it was almost impossible to get fresh milk. We were therefore obliged to be content with condensed. Ice we made for ourselves; in fact, we had to depend on our own exertions for many comforts. We had no screens for the beds. We taught the black boys to make the frames, and covered them ourselves."

"One more question before I go, Miss Nutt. Do you think your West African experience has in any degree seriously affected your health?"

Nurse Nutt smiled. "No, I do not. Although the heat sometimes was terrific, and I felt very 'done up' when I quitted the Niger, the sea voyage quite restored me. It will show you how little I believe the unhealthiness of the climate really injured me when I tell you that as soon as my six months' leave is over I hope to go back again. Can I say more?"

### A Nursing Home at Pekin.

If it were only because the scheme for the establishment of the International Institute of China embraces a Nursing Home for Women it would merit the warm interest and the cordial support of the people of the United Kingdom. For some time the eyes of the world have been upon far Cathay, and the mission of Lord Charles Beresford, resulting in the issue of a report that cannot fail to influence the policy of this and other nations, must tend alike to concentrate and to quicken attention. Let us, by all means, have the open door to trade and commerce. It is desirable for the sake of the common good; it may be essential for the preservation of peace. But, with all the talk about trade following the flag, it is of vital importance that we should not expose ourselves to the charge of purely selfish motives. While we have a clear right to seek the development of China for our material advantage, a clear duty has been imposed upon us to lose no practicable opportunity of improving the social and moral condition of the teeming millions of the Celestial Empire. It may be a mistake for our women to volunteer as missionaries among barbarians who are only too ready to assist them to the crown of martyrdom; but, unless we accompany our plans for exploiting the resources of the vast country for our own benefit by earnest efforts to promote progress and bring about reforms, we shall be false to the principles to which the greatnsss and the growth of the Queen's dominions are primarily due.

How are we most likely to influence the lower classes in China? For, after all, it cannot be doubted that the mandarins really govern the people, and in order to get at the latter effectually it seems requisite in the first place to reach the former. The Rev. Gilbert Reid, an American missionary who has spent nearly two decades of his life among the Chinese, has recognised this need in his plan for the creation of an International Institute in the capital, and has accordingly kindled the hope of achieving a solid and enduring success. Those who know anything of the official classes in China will admit that he has already accomplished much. He has obtained the sanction of the Imperial Board of Foreign Affairs, while Li Hung Chang, Chang Chih Tung, and other Chinese statesmen, are affording the undertaking active individual support. That is to say, they have declared themselves in favour of all that it covers. And it is no paltry project to which Mr. Reid is devoting his time and energy. It includes a library—the first public library in China—and reading-room, where books of reference from the literature of different countries will be obtainable; a museum with four departments—the commercial, the agricultural, the scientific, and the artistic; a lyceum or auditorium, where public meetings may be held and lectures given on historical, scientific, social, moral, and religious questions; class-rooms for carrying on work of the character of a university extension; a social hall or club; separate reception-rooms, where Chinese ladies of better-class families may meet foreign ladies without offending Chinese prejudices, but in harmony with their own sense of propriety; and last, but not least, a nursing home for women. In that portion of the scheme which is intended for the welfare of women Mr. Reid has the valuable help of his wife, who for five years was instructor in a school in Shanghai for high-class girls.

It is not only in China that the enterprise has obtained support. Mr. Reid has enlisted that of all nationalities. On his Advisory Council are the Netherlands Minister, the Consuls-General for Germany and the United States, the heads of the missionary societies, both British and American, the leading traders in Shanghai and Tientsin; though even more significant in such a connection are the names of the president of the Imperial Tung-wen College and the private secretary of Li Hung Chang. Of course money is wanted, and towards the £15,000, the estimated cost of the Institute, and £3,000 a year for the annual expenses, a fair amount has been subscribed. The Chinese have given £3,000, Mr. Thomas Hanbury, a former resident in China, nearly £5,000, the people of America about £6,000, and it is expected that the people of Holland will give £3,000. What remains for the British people to do? Mr. Reid will apparently be satisfied if they will subscribe £3,000, the sum required for the library building. But we suggest that they should also find the money for the nursing home. It has been decided to form a national committee in this country. We regret to say that this step is necessitated by the small financial support which Mr. Reid has received here hitherto. Although he has addressed many meetings, written many letters, and held many conferences, they have produced merely the insignificant total of £250. He charitably attributes the failure to the fact that he has not asked any individual for a contribution, and he thinks that a strong committee may change the complexion of affairs. Lord Loch, Sir G. W. des Vœux (formerly governor of Hong Kong), Sir G. G. Stokes, Professor Max Müller, Mr. William Keswick, M.P. (head of the great firm of Jardine, Matthieson, and Co.), Mr. Pritchard Morgan, M.P., and Sir Cecil Clementi Smith have already joined, and with an organisation embracing these and other representative men at its back, the movement must attract a much wider notice than it has yet obtained. Its features are all excellent, and when once the point is grasped that one of the main objects of the Institute is to break through the barrier which surrounds the mandarins and *literati* of China and infuse into them sound knowledge based on European and American teaching, it may be taken for granted that we shall not hesitate to do our share in the great work. But that share must not be limited to building the library. We must also build the nursing home, and be the means of providing the Chinese with some of the inestimable privileges which are enjoyed by the sick and suffering in the West. Details may be discussed later on, but the scope for the home is obvious. It would surely be a tremendous agency for good, a humanising and an elevating influence. To lose such a chance would be a serious blunder, and it is inconceivable that we shall allow it to slip.

### Appointments.

Bradford Incorporated Nurses' Institution.—Miss S. G. Aitken has been appointed Matron. She was trained at the Western Infirmary, Glasgow, where on the completion of her three years' course she was put in charge of the outdoor patients' department and electrical room, and afterwards was sister for six years of Dr. Finlayson's wards. Miss Aitken has been up to the present night superintendent at the General Hospital, Wolverhampton.

St. Leonard's Infirmary, Shoreditch.—On March 22nd Miss Florence Chambers was appointed Assistant Matron. She was trained at Guy's Hospital, and her subsequent appointments have been night superintendent, Dulwich Infirmary; night sister, Wigan Infirmary; and R.A.E., Indian Army Nursing Service.

County and City of Cork Lying-in Hospital, Cork.—On the 8th inst. Mrs. Mary Blunden was reappointed Matron. She was trained at the North Infirmary, Cork, and at the Rotunda Hospital, Dublin, where she was later staff nurse.

### Where to Go.

Home for Confirmed Invalids, Highbury Terrace, N.—On Wednesday, the 26th inst., at three p.m., the Duchess of Albany will visit the new home and open it. She will also inaugurate a sale of work, and receive purses on behalf of the charity.

### Echoes from the Outside World.

### AN OPEN LETTER TO A HOSPITAL NURSE.

Judging by the crowded state of the Albert Hall on Wednesday afternoon, as well as of the Grafton Galleries on Tuesday evening, not to mention the thousands of bunches of primroses which were worn by men and women everywhere, there does not seem to be any disposition to give up celebrating the anniversary of Lord Beaconsfield's death. The monument in Parliament Square looked quite picturesque with the sun shining upon the wealth of flowers which were massed all round the pedestal, and arranged in any place where it was possible to place them. As usual, the Hong Kong contribution, sent by a rich Greek merchant, attracted a good deal of attention. It was this year in the form of a huge bank of primroses with a screen of ivy leaves at the back, surmounted by an arch and a coronet of the dead statesman's favourite blossoms. An enterprising confectioner has invented a primrose cake, and I should not be surprised if the success he has met with does not induce others to follow his example. It is an ordinary cake, suitable for afternoon tea, which is iced over and then decorated with a wreath of primroses worked out in sugar, the date being introduced in the centre. Saffron cakes are not universally liked, or the colour would be acceptable. How would crystallised primroses do? They might be used as a sweetmeat like the violets, or employed in decorating the cake. I give away this brilliant idea to anyone who may like to utilise it for April 19th, 1900.

---

All who are brought much into contact with Her Majesty are unanimous in saying how very simple and womanly she is in her home life, though, when necessity arises, she can be "every inch a Queen." A remark she is said to have made after the visit of the Sirdar to Windsor Castle was very charming, I think. When Lord Kitchener had left, one of the Ladies-in-Waiting observed, "Your Majesty is, of course, aware that his Lordship is a confirmed woman-hater?" "Is that so?" was the quiet reply. "All I can say is that he was very good and kind to me."

---

We seem to be returning to the practices of the Middle Ages in Italy, when anyone who had an enemy sent a gift of a poisoned ring, a poisoned shirt, or a poisoned glove, and so quietly removed the obnoxious personage out of the way. At present raiment for this purpose seems out of favour, and food is chosen as the medium to convey the fatal dose. A short time ago it was salt with a large proportion of arsenic which a cook introduced into her dishes, so as to ensure the speedy despatch of the clergyman and his family in whose service she had been employed. Then followed the case of a cake sent to an imbecile at an asylum, into which phosphor paste had been so copiously mixed that one person died and several were made very ill. Now from America comes another instance of the rage for what the sensational papers call "romantic poisoning incidents." Miss Cora Tanner, a popular actress, was performing at Omaha, Nebraska. She received a handsome bouquet of flowers and a box of bonbons. Naturally enough, actresses are so much in the habit of receiving gifts from anonymous donors that Miss Tanner's suspicions were in no way aroused. She smelt her flowers and tasted her sweets quite innocently. Unconsciousness quickly supervened, and medical advice had to be hurriedly procured. It is stated, as the result of examination, that both presents were found to be impregnated with prussic acid. Surely the perpetrator of this dastardly attempt at murder must have been seeing or reading a well-known French play where a poisoned bouquet of flowers puts an end to the poor heroine's life.

Talking of theatres, some of you are sure to be wending your way towards the Lyceum—when you get a chance—to welcome back Sir Henry Irving to the theatre which has been the scene of his labours for so many years. On Saturday night the long continued applause which greeted his first appearance gave me quite a lump in my throat. Enthusiasm is wonderfully contagious, and the shouts and cheers, the clapping and the stamping which resounded from all sides of the house, moved one almost as much as a grand burst of song, or the martial roll of drums. As a play, of course, "Robespierre" is beautifully mounted, splendidly acted, and the melodramatic interest of the story well sustained, but the knowledge that the drama is playing sad havoc with history all the time does much, to my mind, to take away the delight of listening. The early love episode with Clarisse de Maluçon, which resulted in the birth of Robespierre's illegitimate son, Olivier, is purely imaginary, and as the Dictator died at the age of thirty-six and his son is here represented as a grown young man, it must have taken place when he was a mere youth. Then the manner of Robespierre's death has been altered to suit the exigencies of the stage. In real life, you will remember, with his jaw shattered from an attempt at suicide, he ascends the scaffold and is guillotined. In M. Sardou's romance, after the Convention has unanimously condemned the man who once had swayed them so absolutely, Robespierre successfully shoots himself and dies on the spot, having first asked and obtained Clarisse's forgiveness. Nor will you find all through much trace of the inhumanity and the mania for the shedding of blood which one has been taught to consider as amongst the chief characteristics of the man whom France once pronounced "incorruptible."

---

You may have seen it announced that a great rush of young men from Finland has commenced. The Czar's manifesto of February 3rd has caused great distress, because, rightly or wrongly, the Finns believe that many of their privileges are to be taken away, and also that whereas they have hitherto been allowed to serve in the Russian army only if they wished to do so, conscription is now to be the order of the day. Rather than submit to being forcibly made into Russian soldiers, many of the young Finns have decided to emigrate. A vessel last week brought 315 men to Hull *en route* for America, and over 200 had arrived previously. A friend who has stayed some months in a boarding-house in Finland tells me that this general exodus of young men, except from a matrimonial point of view, is not nearly so serious as it would be in some lands, where women are less capable than they are in this far northern latitude. Already in the banks in Finland there are more women than men clerks. It is the same at the bureaus where the money is changed. In the postal departments, and even in Government offices women are largely employed, and it is quite as usual to find the post of cashier, accountant, copyist, and translator occupied by a woman as by a man. Most of the Finnish teachers are women, except for certain subjects, and these women count men as well as their own sex amongst their pupils. To an English eye it seems very odd to see a woman sweeping the streets and mending the roads, but this is quite as ordinary an occupation for the lower class women as driving the carts to market and rowing the boats from place to place. The principal sufferers by this departure of so many young men will be the shopkeepers. Strangely enough, in Finland men assistants in shops are distinctly preferred to women, and the number of females to be found behind the counter is very limited. The reason for this, according to one of the large employers of shop labour, is the desire to please his customers, who find male assistants more obliging!

### Tommy: A Hospital Incident.

THE flower of the flock was Tommy, says his mother; only fourteen, and such a good lad. Last week he had earned his first wages—sixpence, in addition to food and board, and that precious sixpence he had carried home on Saturday night "to help with the rent." There are ten children of varying sizes, including a puny, peevish baby which claims a large share of the sickly mother's time and attention, and sixpence represents a valuable addition to the ten or eleven shillings a week earned by the father as an agricultural labourer. This morning Tommy went on to the land with his empty cart to load turnips, sitting on his horse's back, whistling gaily, and flicking with his whip at the trees as he rode under them. Suddenly—no one was there to see how it happened, perhaps he lost his balance, perhaps the horse stumbled and threw him—Tommy was lying on the ground, a kick from the frightened animal had broken his jaw, and the heavy wheel had gone over his chest. So he was found by the next passer-by, who ran shrieking to the farm to say he was dead. The poor old farmer got ready his little cart and pony with trembling hands, put the boy in, and started off for the hospital. Five miles along the rough Fen road they drove at a jog-trot, a bitter east wind blowing; Tommy in his thin old suit, with not an extra wrap round him, propped up there still conscious, occasionally muttering words of the prayers he had learned at Sunday school. At last the little hospital appeared in sight, but by the time kind hands lifted him gently out of the cart to take him to the warmth and comfort within shock and cold had done their work, and kind unconsciousness lulled his anguish.

All that is possible has been done; the doctors shake their heads, and the senior surgeon, with a grave face, strokes the little hand lying on the counterpane, and thinks of his own boys at home. A messenger has gone for the parents, who were in ignorance of the accident, and Tommy lies breathing heavily, and, we think, knowing nothing of what goes on around him. But for a moment his eyes open, the flicker of a smile comes over the little bit of face not hidden by bandages as he says, "Hullo, sister!" and then all is dark to him again. Now the father and mother come in, making no demonstration of grief, not even of surprise, but wearing on their faces that resigned expression which comes of utter weariness, both physical and mental. To them and such as them bad wages and ill-health make the common lot, and dying is one more episode in the grim story of their life. Already two of their little ones have been taken from them; they were too thick upon the ground at home, and Death, with a friendly smile, called them to come where there was more room. The father stands looking at the boy for a few minutes, then, with a heavy sigh, turns away, and sitting down by the ward fire picks up the daily paper and listlessly turns the leaves. His wife sits at the bedside, sometimes telling in a low voice fond anecdotes of Tommy's goodness and sweetness, but for the most part silent, her eyes never wandering from his face. Once, when a gush of blood comes from the injured lung, and the lad fights hard for his breath, her fingers tighten on the arms of her chair, and she moans, "Dear God! My boy!"

Night is come, and with her the messenger. The gas is turned low, and the firelight plays on the blue walls of the ward, and on the white bed where Tommy still lies, higher on the pillows now. Several times this evening he has looked round with recognition in his eyes, and tried to speak, making his mother's heart beat high with hope, but now his breathing gives sure sign that the soul has heard its summons, and is making ready for the journey. Two of Tommy's brothers are here in time to say good-bye; they and the parents kneel round the bed as the chaplain in his clear, steady tones reads the commendatory prayer for the "soul of this Thy servant, our dear brother." Then there is a silence, broken only by a sob from one of the kneeling figures and by each laboured breath of the dying boy. He is lifted yet higher on his pillows, his hand still holds the nurse's, and the quiet voice resumes: "May Christ deliver thee from eternal death, who deigned to die for thee. May Christ, the Son of the living God, place thee for ever within the green and pleasant places of His own Paradise." There is a sudden hush, then a strange little sound from the bed—the blue eyes open wide to take their last look at the things of earth, and Tommy goes to seek and find, instead of the black Fenland, those "green and pleasant places."

### The Nurses' Bookshelf.

[We invite Correspondence, Criticism, Enquiries, and Notes on Books likely to interest Women and Nurses. Address, Editor, THE HOSPITAL (Nurses' Book World), 28 & 29, Southampton Street, Strand, London, W.C.]

AN IDYLL OF THE DAWN. By MRS. FRED. REYNOLDS. (London: James Bowden. 1898.)

SOME two years ago, in reviewing "A Tangled Garden," by Mrs. Reynolds, we prophesied that the writer showed promise of even better work in the future, and our prophecy has, we are happy to say, been realized in the book under notice. In "An Idyll of the Dawn" we are given no plot. The authoress merely endeavours to look back on her childhood, and to see it again through a child's eyes. At the first glance this does not appear a very difficult task, and yet, try as we will, it is hard to recall the hopes and fears and pleasures of our childhood's days when outside the garden gate lay all the wide world—so mysterious and so full of possibilities and adventures. As the "grown-ups" were incomprehensible to us then, so have our childhood's methods of thought and curious wonderings grown incomprehensible to us now. When they are shown us we say, "Oh! how true—how well I remember;" but unless they are shown to us it is to be feared the batterings of a work-a-day world have rendered them to us a closed book. To few is it given to recall and interpret the thoughts of childhood. Quaint as are the conceits in our beloved "Alice," we know full well it is not to his quaint humour that Lewis Carroll owes the appreciation of his many admirers, but to his lifelike delineation of a lovable child's nature and mind. So we must congratulate Mrs. Reynolds on having given us a delightful sketch of two children just as they exist with no straining after effect and with no attempts at "funniness."

The children as they are presented to us in this story live quiet, uneventful lives in their country home, but after the manner of children they surround the every-day events of their lives with a halo of mystery and delight. Their greatest pleasures—and here the authoress strikes a note that shows the artist—are found not in the amusements that are provided for them, but in the accidental ones they provide for themselves. "Now here must be stated a curious fact which someone wise amongst men may possibly account for, namely, that we children, who had our own pony to ride, who, moreover, drove almost daily in our mother's carriage, loathed the latter exercise from the bottom of our hearts, and rejoiced with exceeding joy when, by reason of guests or other just cause, the carriage drove away, leaving us behind. Yet we, the same children to whom a drive in a soft-spring carriage, over fair, smooth roads, brought a feeling of nausea, enjoyed greatly a lift in a hay cart, bumping along over the rough meadows, jerked to this side or to that, and almost as much shaken up and down as were the hay-seeds which kept up a merry dance upon the smooth, dark bottom of the cart."

### A Book and its Story.

### MISS FOWLER'S NEW BOOK.

THE gifted authoress of "Concerning Isabel Carnaby" has produced another book* which sustains her reputation as a writer of unusual brilliancy. Following so speedily on her previous work, it is surprising that "A Double Thread" displays no diminution of the special traits which distinguished it. Here, as in "Isabel Carnaby," smart repartee and epigrammatic dialogue hold naturally first place. A plot one hardly expects to take seriously in a book of this class, but that there is one, and one fraught with grave results to those concerned, is evident to the reader who follows its development.

On a wet December afternoon a small and congenial party of three persons is gathered round the fire in a country mansion. The hostess, Lady Silverhampton, genial and entertaining, and the beautiful Elfrida Harland, an heiress, cynical by circumstance, but proud and affectionate by nature, with Lord Stonebridge as audience, compose the group. The inclement weather outside serves to bring into stronger relief the glow of warmth and comfort reflected from the luxurious surroundings within.

The ball of conversation is being tossed by the two ladies, with an occasional appeal to the audience to arbitrate upon, or to confirm, some startling statement, a difficult and delicate task enough when brilliant and paradoxical badinage is flying.

Lady Silverhampton speaks, gazing out at the rain-bleared landscape, "Rain is always better than snow, because when it rains it is over, while when the snow is over it is only just beginning." Then turning to Elfrida, whose assumed cynicism is the subject of discussion, she says, "It is a fatal mistake for a woman to be cynical." "But," replied Elfrida, "if I don't believe in things it is no use pretending that I do!" "Oh, yes, it is the greatest use in the world. Pretending that you've got a virtue is as good as having a virtue—at least so Shakespeare said, and he was supposed to be a very clever person." Lord Stonebridge mildly interposes that is not a quite accurate rendering of a well-known aphorism. But to be accurate, in Lady Silverhampton's eyes, "is an unpardonable defect," and "to be inaccurate an incurable disease."

One point, at any rate, is clear to her, that the only cure for Elfrida's cynicism is to fall in love. But she, "who was almost perfection," with golden hair shaded with brown and eyes the colour of wild hyacinths, her height a comfortable "three-quarters size," her fortune bequeathed by her grandfather, "an extinct Lord Chancellor," could not accept this remedy for the failing inherited, we are told, from the old Lord Chancellor himself. For from experience she found that men were attracted, some by her wealth, others by her beauty, and "admiration with little love is, as a diet to the human soul, what much stimulant and little food is to the human body." She feels no vocation for falling in love. Lady Silverhampton cannot accept Elfrida's point of view at all. To her to fall in love is perfectly simple. "I fell in love twenty years ago with Silverhampton, and if a woman can fall in love with Silverhampton she can fall in love with anybody. I created a man in my own imagination, and dressed him up in all the qualities that I most admired. . . . He is not in the least like the real Silverhampton, but I adored him." Naturally this process did not commend itself to her guest's critical, fastidious temperament. She rejects with playful scorn the various men suggested by her hostess as suitable *partis*. But she confesses seriously that, although unable to do so, to fall in love might be extremely diverting.

* "A Double Thread." By E. Thorneycroft Fowler. (London: Hutchinson and Co. 6s.)

"I assure you I envy my own scullerymaid when I see her fly up the area steps on Sunday afternoon to meet her young man. It must be delightful to wash one's face with yellow soap to please him."

As a set-off to Elfrida's weariness of her life, in spite of all the good things the Fates had given her, we have the hearty, simple character of Jack Le Mesurier, who, after seven years in India, returns to find everything delightful in England. He comes to join the house party, and after some exchange of playful amenities he and Elfrida are joined by their hostess. "You can't play or sing anything, can you, Captain Le Mesurier?' "No! I cannot perform any parlour tricks, I regret to say." "What a comfort!" exclaimed his hostess, sinking on to a sofa. "I can't bear having people here who can do things; because then they are always wanting to do them, and that is so tiresome for everyone else."

With the introduction of the Welfords, a well-to-do self-centred family of the provincial type, with a son and daughter whose education, or the lack of it, fills them with contempt for their country neighbours, and a mother who "was unexceptionable as a mother but quite uninteresting as a woman," and Mr. Welford himself, a self-made man, of whom "it is only fair to say that the finished article did credit to its manufacturer," comes also on the scene Ethel Harland, passing as Elfrida's twin sister, staying at intervals with her grandparents in the village of Sunningdale, the home also of the Welfords. Captain Le Mesurier is an old schoolfellow of Percy Welford's, and he meets Ethel when staying with him. Being a poor man, although heir-presumptive to a baronetcy, a rich wife is very necessary in his position. But dazzled as he is by Elfrida's brilliancy, he is not blind to the absence of those very charms of "sweetness and cheerfulness" which Ethel possesses. So, "If Elfrida had only been as nice as she looked, Jack would have fallen in love with her; Ethel looked as nice as Elfrida, and seemed even nicer than she looked; therefore, the result of her meeting with Jack was a foregone conclusion."

We are led through the entanglements of the double thread woven by Elfrida to test the character of the man she loves, without suspecting the part she herself is playing to effect her end. For although it is one not unlikely to be played by a complex character, delighting in paradox, such as hers, still it is one open to mistrust and suspicion which only the end in view can condone. The pink diamond incident is an important one in the development of the plot, and leads, perhaps, more than anything to the elucidation of the mystery inseparable from it.

Le Mesurier's uncle, Sir Roger, whom he is to succeed, is a specially entertaining old bachelor. Witty and cynical always, and with Jack as his guest, he is strikingly drawn when at home at his place, Greystone. He has but one desire, that Jack should marry a fortune and Elfrida. Jack agrees to one proviso, but objects, since meeting Ethel, to the other. Here are Sir Roger's views on church-going as a duty to one's rector: "I make it a rule to go to church on a fine Sunday. I consider it a duty due from the squire to the parson." "Certainly, sir," agreed Jack. "So long as there is one member in the family pew it cannot signify who it is. . . . It is equally polite to the church and less fatiguing to the individual." There are other scenes and characters all interesting and charming, particularly those connected with Sir Roger's friend and rector, Philip Cartwright, and Jack's maiden aunt, Camilla Desmond, living in the Deanery, at Silverhampton; but it is impossible to more than briefly touch on the leading characters and incidents, and our desire is to excite, not exhaust our readers' interest in any work reviewed, so that they may read it for themselves without delay.

### Everybody's Opinion.

[Correspondence on all subjects is invited, but we cannot in any way be responsible for the opinions expressed by our correspondents. No communication can be entertained if the name and address of the correspondent is not given, as a guarantee of good faith but not necessarily for publication, or unless one side of the paper only is written on.]

### A DEARTH OF TRAINED NURSES.

"A SUPERINTENDENT OF NURSES" writes under this heading as follows: Will you kindly allow me to draw attention to the difficulty the old established nursing institutions have in getting trained nurses to join them; and to the fact that nurses, as a rule, prefer to form small establishments where they can take their own fees. Often in these small establishments there is no matron or person in authority, and each nurse goes out in turn, quite regardless of the fact that another might be better able to undertake the nursing of a particular case. Surely this is to be regretted, as it will not only tend to lower the standard of nursing, but will increase the difficulty of obtaining suitable nurses for special cases. If nurses are to retain for their profession the important position it has gained, they ought to remember that strength can only be attained by union. Most of our institutions are not worked for personal gain, but with the object of securing for the public the best possible class of nurses, and for the nurses the full amount of their earnings, less working expenses. This seems to me the most satisfactory plan, as it insures to the nurse a comfortable home, as well as care and a continuance of salary during sickness, besides giving them a social standing, and is a guarantee to the public that the nurse has received a proper course of training. I write this in the hope that some persons who are interested in the older private nursing associations may communicate with you, and suggest: (1) What are the causes of the reluctance of nurses to join these Homes? (2) The remedies most likely to be of use in retaining their services?

### BRONCHITIS KETTLES.

"MEDALLION 50,320" writes: May I, although only an amateur nurse, call your attention to the great danger incurred in using an ordinary bronchitis kettle? For many years, during various throat and lung complaints which came to a successful issue, I used a kettle from the kitchen with an improvised long spout of cardboard or brass curtain tube. Having lost, under unutterably sad circumstances, a child of five from membranous croup, I provided myself with a proper steam kettle in readiness for the next occasion on which our doctor might order steam. For this murderous article I paid 3s. 6d. No word of caution was given to me by the shopman of whom I purchased it, and not being at all a scientific person I naturally filled the kettle with water when I required it, screwed the nozzle on tight, and placed it on the fire, expecting, of course, to shortly find the air being impregnated with steam. As I sat, perhaps a little sleepy after a hard day's work, I was startled by an extraordinary noise from the fireplace, and a quantity of boiling water was projected from the kettle with such force that it struck the opposite wall. Had it not been that, with the almost unconscious caution arising from the rearing of a large family, I had turned the spout of the kettle a little away from the bed on which my child lay, a severe and perhaps a blinding scald would have been added to the broncho-pneumonia from which she was suffering. A relative of mine had a similar experience, but in her case her boy's bed was on the opposite side of the room to the fireplace, and although the boiling water was projected on to the bed it did not reach the patient's face. Had it done so, the child, a peculiarly sensitive little fellow, would in all probability have died from the shock. A year or so ago an inquest was held on a child who was scalded to death, his mother having directed the spout over the cradle. As one is sometimes ordered to make a sort of tent over a patient's bed or cot, and to direct the vapour under it, I should be glad of information on the subject. The ordinary bronchitis kettles of commerce have only one outlet for water or steam when the nozzle is screwed on—the long spout—but in the picture of a bronchitis kettle given in the St. John Ambulance Nursing Handbook there is what I presume to be a safety-valve at the back of the kettle. For the benefit of us poor, ignorant mothers could not some enterprising firm devise a kettle with a ridge, beyond which the amateur is advised not to let the water rise?

### For Reading to the Sick.

"CONSIDER the lilies of the field, how they grow; they toil not, neither do they spin.'—*St. Matt.* vi. 28.

TO THE SNOWDROP.

Thou first-born of the year's delight,
Pride of the dewy glade,
In vernal green and virgin white,
Thy vestal robes arrayed. —*Keble.*
Sweet nurslings of the vernal skies,
Bathed in soft airs and fed with dew;
What more than magic in you lies,
To fill the heart's fond view?
In childhood's sports, companions gay,
In sorrow, on life's downward way,
How soothing, in our last decay,
Memorials prompt and true.

Ye dwell beside our paths and homes,
Our paths of sin, our homes of sorrow,
And guilty man, where'er he roams,
Your innocent mirth may borrow.
The birds of air before us fleet,
They cannot brook our shame to meet;
But we may taste your solace sweet,
And come again to-morrow.

Alas! of thousand bosoms kind
That daily court you and caress,
How few the happy secret find
Of your calm loveliness!
"Live for to-day, to-morrow's light
To-morrow's cares shall bring to light;
Go sleep like closing flowers at night
And heaven thy morn will bless."

**Reading.**

All life in Nature is the gift of the Spirit, and manifests His hidden presence. It is the Spirit who has beautified and garnished all the works of nature. He has ever been at work in the material world, evolving, by His presence, form, beauty, order, life.

"The Spirit of God," says Ruskin, "is around you, in the air that you breathe, His glory in the light that you see, and in the fruitfulness of the earth, and the joy of its creatures. He has written for you, day by day, His revelation, as He has granted you, day by day, your daily bread."

If we would know the secret of heavenly-mindedness, of Christ-like uprightness, purity, gentleness, truthfulness, self-sacrifice, and charity, we must confess the sanctifying agency of the Holy Spirit, inviting the souls of men to Jesus Christ, and enabling them to follow this perfect example It is only when men yield to and co-operate with the blessed influences of the Spirit of God, that others will "take knowledge of them, that they have been with Jesus."—*From "Natural Religion."*

Make Thou my spirit pure
As are these frosty skies,
Or this first snowdrop of the year
That in my bosom lies.
As these white robes are soiled and dark
To yonder shining ground;
As this pale taper's earthly spark
To yonder argent round!
So shows my soul before the Lamb,
My spirit before Thee,
So in my earthly state I am
To what I hope to be!
Break up the heavens O Lord! and far
Thro' all yon starlight keen,
Draw me, Thy bride—a glittering star
In raiment white and clean.
—*Tennyson.*

### Travel Notes.

By Our Travelling Correspondent.

### XIX.—MENTONE.

After an absence of eight weeks we are again on the Riviera, making one more step along those enchanting shores. Considered broadly, the Riviera seems always to hold its place as first favourite with invalids and those who, without quite coming under that category, like to make assurance doubly sure by spending the cold weather away from our treacherous shores. Egypt and Algiers are becoming very popular, but still the lovely Corniche holds its own, the chief reason being the variety of its scenery and the facilities for choosing different temperatures and other climatic advantages for different states of health.

### Expenses of the Journey.

Viâ Dover, Calais, Amiens, and Paris the fare is £7 17s. first class, and £5 7s. 1d. second class. Viâ Dieppe it is £6 14s. 7d. first class, and £4 13s. 2d. second. Against that gain you must put the fact that you must drive across Paris, cab fare 2fr. 50 without luggage, and with it I find I generally have to pay 4 fr. if I desire to escape much altercation. Moreover, one loses the immense advantage and comfort of registering the luggage through by the Ceinture Railway.

### Where to Break the Journey.

If you go by the Dieppe route, and second class, you cannot get straight through, but if you think it advisable to sleep in Paris (and the journey already will have been fairly long) you can go on the next day by the train from the P.L.M. station at 2 p.m., reaching Mentone at 10.39 the next morning. By adopting this route you can sleep at Lyons, which is reached at 10.53, just in time for a good night's rest (Hotel Collet and Continental), going on the next morning at 10.18 a.m., reaching Marseilles at 6.4 p.m., sleeping at the Terminus, adjoining the station. Second class trains are not convenient after Marseilles, and you will have to go on at an early hour the next morning; or it is a good plan to take second-class tickets to Marseilles and change to first from there. This gives you a much better choice of trains.

### Climatic Advantages of Mentone.

The inhabitants of Mentone claim for it the privilege of being the mildest spot on the Riviera, and probably, with the exception of the little spot around Beaulieu, it is so. Indeed, Nature proves the statement, for lemons flourish at Mentone in large quantities, and they are far more delicate and susceptible to the slightest cold than the more hardy orange. Dr. Bennett first brought the virtues of Mentone before the English public. He was an old friend of my father, and one of my earliest recollections is that of listening to what my childish mind considered his wearisome prosings about the climate and advantages of Mentone, so that long before I ever saw it I seemed instinctively acquainted with its topography, its winds, temperature, and general attractions. The form of the bay on which the town is built protects it from north, east, and west winds, and causes it to be flooded with the sun on the south, while the mountains which protect it from the northern blasts also act in some mysterious way upon the moist breezes driven in from the sea. These sea breezes, unable to pass the mountains, become condensed and hang as a slight haze over the bay in the hottest hours of the day. This softens the atmosphere, and certainly many invalids with an irritable cough seem to do better at Mentone than at other places along the shore. Consumption and heart disease both yield to the bland air of this charming spot, and it is peculiarly beneficial to cases of bronchitis and to those who very readily take cold. I have seen life prolonged many years in a case of advanced pulmonary consumption aggravated by heart disease. The mistral or north-west wind is but little felt, which is certainly a strong point in favour of the place.

### Hotels and Apartments.

Life is distinctly not so expensive as at Nice or Cannes, and if means are limited it is quite possible to live at Mentone on a small income. The hotels are so numerous and so good that it is difficult to make a choice. The town is rather straggling and consists of two parts, the west towards Cap Martin and the east called Garevan. At the west end, there is the Hotel Cap Martin, luxurious and rather expensive room from five francs, dinner seven francs. The Gorbio Hotel, a mile from the town, a trifle less expensive and very agreeable. There are alsc the Iles Britanniques, the Hotel du Louvre, the Prince de Galles, the Splendide, the Windsor, &c. On the Garavan side are the Bellevue, Hotel des Anglais, and the Grand Hotel. The Britannia, near to the Garavan Station, is very moderate. At most of these you can make an arrangement for pension from eight francs, but the rooms would not have a southern aspect. If an invalid is able to be much out of doors, this is not always essential, though it is, of course,

A Mentone Mule Equipped Against the Sun.

most desirable. There are several excellent hotel pensions. The Pension Santa Maria I can recommend from personal experience. Villas are to be had at fairly moderate rates; application should be made to Monsieur Isnard, house agent. Apartments are rather scarce, though they are to be had, and for a long stay for several people it may be worth while. But I generally recommend hotels. In the end one saves little if anything by taking rooms, and it involves a good deal of trouble.

### Churches, Trams, Amusements.

There are two English churches, Christ Church and St. John's, and a Presbyterian Church. The Roman Catholic Church of St. Michel is on the apex of the conical group of buildings which form the old town. To climb up to it is an interesting walk, and the view from the platform on which it stands is beyond description lovely; the old town is very singular and interesting, formed of narrow streets furnished with steps like Roccabruna, up and down which the mules step daintily. The sun has such power in this sheltered spot that these useful quadrupeds are furnished with large straw hats. Their ears project through holes at the sides, the crowns are trimmed with scarlet and blue braid, and altogether they present a most comical appearance. A convenient tram runs from one end to the other of the town every ten minutes, and diligences go to Ventimiglia along the Corniche twice a day. Mentone is not so gay a place as Nice or Cannes, and for that reason it is preferred by many; it is, however, by no means dull. There is a club with a very moderate subscription—60 francs for the season—a casino, and a theatre, and occasional balls are given. It is splendidly placed for excursions into the surrounding country, and there are excellent carriage roads. Of these excursions I shall hope to speak next week.

### TRAVEL NOTES AND QUERIES.

ROME (Knight Errant).—The water of Rome is very good, and the sanitation, since so many English and Americans have gone there, on the whole excellent. No, I do not think less than a two months' stay there is worth considering. The journey s expensive. The cheapest route, viâ Newhaven and Dieppe, first-class, is £8 19s. 7d.; second, £6 5s. 5d. The second is quite comfortable, and suitable for any but invalids. If you are strong you can go through with only one night's rest, at Milan; if not, you must sleep also in Paris.

THE ARDENNES (Cyclist).—The roads in the Southern Ardennes are excellent for cyclists south of Namur. About Dinant they are very good, though not equal to those in France. There are, however, not many hills, for though it is a well-wooded and by no means flat country, the main roads avoid the hills and skirt their feet. In Belgium cycling is not ideal, there are so many miles of pitched roads.

DINANT (Stella).—There are now several good hotels. From long habit I prefer the Tête d'Or, but there are others as good. Pension from 7½ francs. Hôtel des Postes have the same terms, but if you want something still cheaper try the Hôtel des Familles or the Hôtel de l'Europe. 2. Yes, the church is very fine. Be sure to see it in the gloaming, when the only lights are those twinkling before the altar.

SOUTH OF FRANCE (Rondinella).—I think you could not do better than St. Jean-de-Luz, close to the Spanish frontier. It is a long journey and consequently expensive, but living there is remarkably cheap. Second class, viâ Paris and Bordeaux, £4 5s. 6d.; viâ Newhaven and Dieppe, £3 13s. 7d. Pension terms at the Hôtel de Poste or Hôtel France, 7 frcs. per day. Apartments are very reasonable, and with a femme de ménage you will manage well and very cheaply. I spent four months there last year, and derived much benefit from the sunshine and bracing air. Let me hear again if I can help you further. You were too late for this week's issue.

*(For Travel Advertisements see Page* xviii.*)*

### Ceremony at the Curragh.

A VERY interesting event in connection with the nursing branch of the Soldiers and Sailors Families' Association has just taken place at the Curragh Camp. Quite recently the Princess of Wales expressed a wish that in future all nurses working entirely for the Soldiers and Sailors' Families' Association should be known as Alexandra nurses, and under this new designation Nurse Diamond was the first to receive public recognition for her really excellent services, which have been carried on for over six years amongst the soldiers' wives and families in the Curragh. Viscountess Downe presided at the meeting, made a short speech, and read a congratulatory telegram from the council of the S.S.F.A.; after which Miss Combe, as president of the Curragh branch of the association, presented Nurse Diamond with a silver medal, which bears an excellent likeness of the Princess of Wales on the obverse and the name of the association on the reverse.

### Minor Appointments.

CARSHALTON COTTAGE HOSPITAL.—Miss Helena Kemp has been appointed Nurse-Matron of this hospital, which will shortly be opened by Lord Rosebery. Miss Kemp was trained and later sister of the Grimsby and District Hospital. Her subsequent experience has been in the Victoria Hospital for Children, and in private nursing abroad and at home.

SHEFFIELD UNION INFIRMARY.—On April 5th Misses Rhoda Harvey and Kate Mary Cutler were appointed Charge-Nurses here. Miss Harvey was trained at the institution, and Miss Cutler was trained and later charge-nurse at Mill Road Infirmary, Liverpool.

MALLING UNION WORKHOUSE.—On March 22nd Miss Jessie Ryal, of the Park Hospital, Hither Green, was appointed Superintendent Nurse.

### Notes and Queries.

The contents of the Editor's Letter-box have now reached such unwieldy proportions that it has become necessary to establish a hard and fast rule regarding Answers to Correspondents. In future, all questions requiring replies will continue to be answered in this column without any fee. If an answer is required by letter, a fee of half-a-crown must be enclosed with the note containing the enquiry. We are always pleased to help our numerous correspondents to the fullest extent, and we can trust them to sympathise in the overwhelming amount of writing which makes the new rules a necessity.

Every communication must be accompanied by the writer's name and address, otherwise it will receive no attention.

*Lady Warwick's Home.*

(28) Will you please tell me whether ladies are trained as nurses in Lady Warwick's Home for Crippled Children, and if so, how are they enabled to find out when vacancies for probationers occur?—*Catherine.*

Write to the Matron of the Countess of Warwick's Home for Crippled Children, Avon House, Emscote, near Warwick, for the information you require.

*Soldiers' Widows.*

(29) Could you kindly tell me if anywhere in England there is a home for soldiers' widows (non-commissioned officers)?—*Nurse.*

Apply for information to the Secretary of the Soldiers and Sailors Families Association, 23, Queen Anne's Gate, Westminster, and to the Secretary Royal Cambridge Asylum for Soldiers' Widows, 20, Cockspur Street, S.W.

*Bath.*

(30) You have very kindly helped me before, and I should be so glad if you would tell me if there is a home at Bath where a lady, who is very poor, and suffering from rheumatism, could be received for treatment free of charge or for a small sum weekly. It is a very deserving case.—*F. H.*

The Royal Mineral Water Hospital, Bath, is open to the poor of the United Kingdom. A medical certificate, and a certificate of poverty only are needed. Write to the secretary.

*Midwifery Fees.*

(31) Can you please tell me what is the usual fee paid in London to an obstetric nurse who is nursing on her own account?—*M. L.*

From £5 5s. to £8 8s. a month.

*Spinal Case.*

(32) Could you kindly inform me if there is any hospital or home which would receive a young woman—a spinal case—for a year? The doctor has told her that she will have to keep in bed for that period.—*District Nurse.*

You might try the Yorkshire Home for Chronic and Incurable Diseases, Harrogate. If some payment could be made some cottage hospital whose beds are only partly occupied might be willing to take such a case.

*Nursing Homes in Glasgow.*

(33) Will you be so good as let me know the names and address of "nursing homes" in Glasgow?—*Nurse J. B.*

See "The Nursing Profession: How and Where to Train." (The Scientific Press. 2s.) You will find there all the information you require.

*The Nurses' Co-operation.*

(34) Can you give me any information about the Co-operative Nursing Association? Do you know if they have a branch in Manchester? Would a nurse with two years' certificate be eligible to become a member.—*J. S.*

If by the Co-operative Nursing Association you mean the Nurses' Co-operation, 8, New Cavendish Street, W., they have no branches. A *three* years' certificate is an essential qualification.

*Home for Incurable and Wife.*

(35) I shall be much obliged if you will let me know some inexpensive boarding-house or home, suitable for a poor man (suffering from an incurable internal complaint) and his wife, at Bournemouth? They are able to pay a reasonable sum, being respectable shopkeepers.—*C. M. P.*

If you apply to the Secretary, the Provident Infirmary and Cottage Hospital, Bournemouth, he would probably be able to help you.

*Maternity.*

(36) Will you kindly give me address of Royal Maternity Society? or (2) of any society where I could apply for work as district midwife?—*Adelaide D.*

The address of the Royal Maternity Charity is 31, Finsbury-square, E.C. 2. See advertisement in our columns.

*Hong Kong.*

(37) Please tell me to whom I should apply for nursing in Hong Kong.—*China.*

The Matron, Government Civil Hospital, Hong Kong; the Secretary of the Colonial Nursing Association, Imperial Institute, W., might be able to give you some information.

*Home for Dipsomaniacs.*

(38) Please can you tell me of a home or institute for a lady who suffers from intemperance, and is anxious to be cured?—*Snider.*

Lady Henry Somerset receives ladies at her Industrial Farm Colony for Inebriate Women at Duxhurst, Surrey.

*Epileptic.*

(39) Could you kindly tell me of any home or institution where a boy of eight years could be admitted? He has had epileptic fits from a year old. He is also dumb, and unable to walk through paralysis of legs. His parents are only farm labourers, and could not afford to pay. I should be very glad if you could tell me of any institution that is free.—*Nurse B. (Notts).*

Write to the Secretary of the County Asylum, Shenton, Notts, and state the circumstances of the case.

## Figures and Tables

**Figure f1:**